# Psychology Applied to Medicine

**Published:** 1907-06

**Authors:** 


**Psychology Applied to Medicine.** Introductory Studies by **David W. Wells,** M.D. Lecturer on Mental Physiology, and Assistant in Ophthalmology, Boston University Medical School; ophthalmic Surgeon, Massachusetts Homeopathic Hospital, Boston; oculist, Newton (Mass.) Hospital. Philadelphia: F. A. Davis and Company. 1907. Price, $1.50.

The study of physic phenomena is always a most interesting one and is a necessary adjunct to the complete understanding of the normal or diseased mind. This work of Doctor Wells is an essay—elaborated into a series of studies—pertaining to mental phenomena—as "habit, instinct, reason, sensation, hypnosis, psycho-therapeutics and the psychic element in medicine. To those interested in psychic phenomena, it is a very valuable, concise and interesting little work. To those not interested, it may be the means of encouraging an appetite for these studies. It is well written, well printed, and well worth a careful perusal.

